# Trend and Correlates of Leadership Competencies Among Female Health Professionals in Albania

**DOI:** 10.3389/fpubh.2019.00109

**Published:** 2019-04-30

**Authors:** Klevis Caushaj, Katarzyna Czabanowska, Enver Roshi, Herion Muja, Genc Burazeri

**Affiliations:** ^1^Department of International Health, School CAPHRI (Care and Public Health Research Institute), Maastricht University, Maastricht, Netherlands; ^2^Operational Department, American Hospital, Tirana, Albania; ^3^Faculty of Health Sciences, Institute of Public Health, Jagiellonian University, Krakow, Poland; ^4^Department of Public Health, Faculty of Medicine, University of Medicine, Tirana, Albania

**Keywords:** Albania, competency level, competency gap, female health professionals, leadership competency, public health leadership, Western Balkans, women

## Abstract

**Aim:** Our aim was to assess the trends and correlates of the leadership competency level of female health professionals in Albania, a transitional country in the Western Balkans, based on a standardized international instrument.

**Methods:** Two nationwide cross-sectional studies were conducted in Albania in 2014 (first wave; *n* = 105 women) and subsequently in 2018 (second wave; *n* = 121 women). A structured questionnaire was administered to all female participants aiming at self-assessing the *current* level of leadership competencies and the *required* (desirable) level of leadership competencies for their current job position. The questionnaire consisted of 52 items pertinent to eight domains. Answers for each item of the instrument ranged from 1 (“minimal competency level”) to 5 (“maximal competency level”). Overall summary scores (range: 52–260) were calculated for both the current and the required leadership competency levels in both survey rounds, based on which the gap in leadership competency level was also computed (required *minus* current competency level). Binary logistic regression was used to assess the correlates of the gap in leadership competency level among study participants.

**Results:** In multivariable-adjusted logistic regression models, there was evidence of a positive association between the gap in leadership competency level and: workplace in urban areas (OR = 3.2, 95%CI = 1.6–6.6); work experience (OR_[for 1 year increment]_ = 1.1, 95%CI = 1.0–1.2); first round of the survey conducted in 2014 (OR = 2.1, 95%CI = 1.0–4.3); and, particularly, a high managerial job position/level (OR = 3.8, 95%CI = 1.6–9.3). Conversely, there was an inverse relationship with the age of women (OR_[for 1 year increment]_ = 0.9, 95%CI = 0.8–1.0).

**Conclusion:** Our study provides useful evidence about trends over time and selected correlates of the gap in leadership competencies among female health professionals in Albania. Policymakers and decision-makers in Albania and other countries should be aware of the unmet need for leadership training of female health professionals at all levels.

## Introduction

Several competency frameworks have been already established aiming at addressing public health leadership and medical leadership competencies in different countries (1–4). A more recent framework was established by Czabanowska et al. (5), in the context of the Leaders for European Public Health (LEPHIE) Erasmus Multilateral Curriculum Development Project, supported by the European Union Lifelong Learning Programme (5). This framework is recommended for use in all countries of the European Region. In particular, the goal of this newly introduced instrument is to promote a competency-based European public health leadership curriculum and ultimately strengthen the leadership knowledge, abilities, skills, and competencies among public health professionals operating at all levels and in different European countries (5).

Albania constitutes a transitional country in the Western Balkans, which has been characterized in the past few decades by rapid political and socioeconomic changes which have been linked to negative health outcomes (6, 7). Of note, there has been a significant change in the epidemiological profile of the Albanian population in the past 30 years with a remarkable transition toward non-communicable diseases (NCDs) (8). As a matter of fact, there is a huge increase in the total burden of NCDs in Albania including cardiovascular diseases, cancer, lung and liver diseases, and diabetes (8). However, the current health workforce is not capable of managing properly this multi-factorial and complex burden of disease in Albania (9). Therefore, based on the rapid epidemiologic transition, there is an urgent need to improve and strengthen workforce abilities and competencies including also leadership capacities in order to address the current health challenges of the Albanian population and meet the objectives of the health care reform.

Importantly, the Albanian health system suffers from a severe shortage of health professional including also the public health workforce. The health personnel per capita ratio has been reported at about 1.2/1,000 for doctors and 3.6/1,000 for midwives/nurses (9). More importantly, human resources in health care services are characterized by an unequal distribution (10). Hence, medical specialists are mainly concentrated in Tirana and in the other large cities of Albania, whereas small districts and especially the remote areas of the country experience a severe shortage of health care personnel and public health professionals. Nonetheless, a main achievement is the establishment of the continuing education system (CES) for health professionals in Albania including physicians, dentists, and pharmacists. The successful completion of the first CES round provided an opportunity to improve this experience and expand the ongoing education also among nurses and midwives (9). Yet, further efforts are envisaged to standardize their professional level, motivation, and distribution according to the skills and competencies in the workplace.

However, demographic trends and epidemiological profile of the Albanian population require a workforce of health professionals and especially public health practitioners with new knowledge, abilities, skills, and competencies. Besides filling in the gaps in health-care professionals and public health specialists created over the years, an effective human resources strategy should ensure the restoration of authority and dignity of health workers and public health professionals in Albania (9). In particular, efforts should be made for ensuring a proper distribution of the health workforce and public health specialists in all regions of Albania (9) and improving their training and qualification, including also leadership competencies.

In this framework, the aim of our study was to assess the trends and correlates of the leadership competency level of female health professionals in Albania, a post-communist country in South Eastern Europe, based on a standardized international instrument. We hypothesized a bigger self-perceived gap in leadership competency level among women working in urban areas and/or those promoted in high managerial positions given the higher pressure and demands for high-quality services.

## Materials and Methods

The current analysis was based on two survey rounds conducted in 2014 (first wave/round) and subsequently in 2018 (second wave/round).

Both rounds of the survey consisted of cross-sectional studies applying the same methodology for sampling of health professionals and employed the same instrument for data collection (5), which had been already validated in Albania in May 2014 (11), before conducting the first round/wave of the survey.

The survey conducted in July-December 2014 targeted a nationwide representative sample of 120 female health professionals working at different health institutions all over Albania [primary health care services (*n* = 40), regional hospitals (*n* = 40), University Hospital Center “Mother Teresa” (*n* = 10), Institute of Public Health (*n* = 10), Regional Directorates of Public Health (*n* = 10), and Health Insurance Fund (*n* = 10)]. The sampling frame was made available from each institution. Based on the sampling frame, a random sample of health professionals was drawn from each institution. Of 120 targeted individuals, 105 female health professionals participated in the study (response rate: 88%). Details about the first survey round have been also described elsewhere (12).

Conversely, the second round of the survey conducted in June-November 2018 targeted a similarly nationwide representative sample of 150 female health professionals working at the same health institutions at both central and local level in Albania [primary health care services (*n* = 50), regional hospitals (*n* = 50), University Hospital Center “Mother Teresa” (*n* = 10), Institute of Public Health (*n* = 15), Regional Directorates of Public Health (*n* = 15) and Health Insurance Fund (*n* = 10)]. Of 150 targeted female health professionals, 29 individuals did not participate (11 women were willing but did not manage to answer, whereas further 18 women refused to participate). Therefore, the study sample in 2018 consisted of 121 female health professionals, with an overall response rate of: 121/150 = 81%.

In both survey rounds, a structured self-administered questionnaire was applied aiming at self-assessing the *current* level of leadership competencies and the *required* (desirable) level of leadership competencies for the actual job position of female health professionals.

The questionnaire comprised 52 items categorized into the following eight competency domains (subscales) (5), each of which matching one educational session within public health leadership curriculum (5, 13): (i) systems thinking; (ii) political leadership; (iii) collaborative leadership: building and leading interdisciplinary teams; (iv) leadership and communication; (v) leading change; (vi) emotional intelligence and leadership in team-based organizations; (vii) leadership, organizational learning, and development, and; (viii) ethics and professionalism.

Domains (subscales) of the framework were a result of a systematic review process constituting an important attempt to define, profile, and position public health leadership through an analytically developed, comprehensive and multidisciplinary competency framework (5). As convincingly argued elsewhere (5), domains of this framework promote a collaborative and shared leadership and embrace specific public health leadership attributes such as the ability to identify and engage stakeholders in interdisciplinary projects to improve public health; to ensure that organizational practices are aligned with changes in the public health system and the larger social, political, and economic environment and ability to build alliances, partnerships and coalitions to improve the health of the populations (5).

Answers for each item of each subscale of the instrument ranged from 1 (“minimal competency level”) to 5 (“maximal competency level”).

An overall summary score (range: 52–260) and a subscale summary score for each domain were calculated for both, the current level of competencies and the required level of competencies, in each of the two rounds of the survey.

Furthermore, the gap between the required (desirable) and the current level of leadership competencies was calculated for each participant, as a difference between the summary score of the required (desirable) level and the current level of leadership competencies. In the analysis, the competency gap was dichotomized into: “competency gap” (all positive summary score differences between the required and the current level of leadership competency levels) vs. “no competency gap” (including zero and/or negative scores).

In addition to the leadership competency tool, the (structured) questionnaire inquired about demographic information (age of female health professionals), place of work (urban areas vs. rural areas), type of diploma, and main degree obtained (which in the analysis was dichotomized into: health sciences [including medicine, public health, nursing, pharmacy, and dentistry] vs. other diploma/degree [including law, economics, social sciences, engineering, or other diplomas/degrees]), years of working experience, training in leadership skills (dichotomized into: yes vs. no), as well as the current job position (which is the analysis was categorized into: high, middle and low managerial position/level).

In both survey rounds, the internal consistency for the current and the required level of leadership competencies was assessed by use of Cronbach's alpha (14). Conversely, Fisher's exact test (for categorical variables) and Mann-Whitney's test (for numerical variables) were used to compare the distribution of baseline characteristics between female health professionals involved in the two survey rounds. Also, Mann-Whitney test was used to compare the overall scores and the subscale scores of the current and the required level of leadership competencies between females participating in the two waves of the survey. General linear model was used to calculate the crude (unadjusted) mean values and their respective 95% confidence intervals (95%CIs) and *p*-values for the gap in leadership competencies level (calculated as follows: summary score of the required level *minus* summary score of the current level) between women included in the two survey rounds (2014 vs. 2018). On the other hand, binary logistic regression was employed to assess the correlates of the gap in leadership competency level (dichotomized into: “competency gap” vs. “no competency gap”) among female participants. Crude (unadjusted) odds ratios (ORs: competency gap vs. no gap) and their respective 95%CIs and *p*-values were calculated. Subsequently, multivariable-adjusted ORs, 95%CIs and *p*-values were calculated in logistic regression models controlling simultaneously for all covariates [age of participants (numerical variable), years of working experience (numeric), place of work (urban vs. rural areas), job position (low, middle and high position/level), main degree (health sciences vs. other degrees), training in leadership skills (yes vs. no), and round/wave of the survey (2014 vs. 2018)]. All the logistic regression models met the goodness-of-fit criterion as appraised by the Hosmer and Lemeshow test (15).

For all statistical tests performed, a *p* ≤ 0.05 was considered as statistically significant. All statistical analyses were performed by use of the Statistical Package for Social Sciences (SPSS, version 19.0).

## Results

Mean age of female health professionals included in the first round (conducted in 2014) was higher than in the second round of the survey (in 2018): 44.4 ± 9.9 years vs. 40.8 ± 8.9 years; *P* < 0.01 (Table 1). About 28% of participants in 2018 were working in rural areas compared with only 14% in 2014 (*P* = 0.02). About 78% of the women in 2018 had their main degree in health sciences compared with 87% in the first round, a difference which was only borderline statistically significant (*P* = 0.09). In 2018, mean working experience was 13.2 ± 9.8 years, whereas in 2014 it was 19.0 ± 9.6 years, a difference which was highly statistically significant (*P* < 0.01). On the other hand, 33% of female health professionals included in the 2018 round had a high managerial job position compared with only 21% of their counterparts involved in the first round conducted in 2014, notwithstanding the lack of statistical significance of this finding (Table 1).

**Table 1:** Baseline characteristics in two nationwide representative samples of female health professionals in Albania, in 2014 and 2018.

**Characteristic**	**2014 sample (*N* = 105)**	**2018 sample (*N* = 121)**	***P***
**Age (years)**	44.4 ± 9.9^*^	40.8 ± 8.9	0.004^‡^
**Place of Work:**			
Urban areas	90 (85.7)^**†**^	87 (71.9)	0.015^**¶**^
Rural areas	15 (14.3)	34 (28.1)	
**Main Degree:**			
Health sciences	91 (86.7)	94 (77.7)	0.086^**¶**^
Other	14 (13.3)	27 (22.3)	
**Work experience (years)**	19.0 ± 9.6	13.2 ± 9.8	<0.001^‡^
**Job Position:**			
High managerial level	22 (21.0)	40 (33.1)	0.114^**¶**^
Middle managerial level	58 (55.2)	54 (44.6)	
Low managerial level	25 (23.8)	27 (22.3)	

* *Mean values ± standard deviations*.

† *Numbers and column percentages (in parentheses)*.

‡ *Mann-Whitney test*.

¶ *Fisher's exact test*.

For the year 2014, the internal consistency of the overall scale of the leadership competency instrument (52 items) was Cronbach's alpha = 0.88 for the current competency level and Cronbach's alpha = 0.96 for the required competency level; for the year 2018, these values were alpha = 0.81 and alpha = 0.95, respectively (data not shown in the tables).

Mean value of the summary score (52 items) for the current level of leadership competencies was significantly lower in 2018 than in 2014 (138 ± 10 vs. 143 ± 12, respectively; *P* < 0.01) [Table 2–upper panel]. In particular, “political leadership” and next the “collaborative leadership” subscales' scores were significantly lower in the second round (in 2018) compared to the first round (in 2014) of the survey. As for the required (desirable) level of leadership competencies, there was evidence of a bigger difference between the two survey rounds: the overall score was 141 ± 22 in 2018 as opposed to 154 ± 26 in 2014 (*P* < 0.01). There were significant differences for all subscale summary scores for the required level of leadership competencies between the two survey rounds (all *P* < 0.02) [Table 2–lower panel].

**Table 2:** Summary score of each domain (subscale) of the “leadership competency instrument” for the *current* and the *required* level of competencies in two nationwide representative samples of female health professionals in Albania in 2014 and 2018.

**Domain (subscale)**	**Mean values ± standard deviations**		*P*-value^*^
	**2014 sample (*N* = 105)**	**2018 sample (*N* = 121)**	
**Upper Panel: Current Level of Competencies:**			
Overall scale *(52 items)*	143.2 ± 12.4	138.0 ± 10.1	0.001
Systems thinking *(7 items)*	21.2 ± 2.4	21.4 ± 3.1	0.943
Political leadership *(8 items)*	22.1 ± 6.0	19.4 ± 4.8	0.001
Collaborative leadership: building and leading interdisciplinary teams *(5 items)*	12.2 ± 3.0	11.3 ± 2.9	0.005
Leadership and communication *(7 items)*	16.9 ± 2.2	16.2 ± 2.1	0.024
Leading change *(6 items)*	17.7 ± 2.2	17.5 ± 2.2	0.642
Emotional intelligence and leadership in team-based organizations *(6 items)*	18.5 ± 2.5	18.4 ± 2.5	0.475
Leadership, organizational learning and development *(7 items)*	16.9 ± 2.0	16.4 ± 2.1	0.066
Ethics and professionalism *(6 items)*	17.7 ± 2.1	17.5 ± 2.0	0.515
**Lower Panel: Required Level of Competencies:**			
Overall scale *(52 items)*	154.1 ± 26.2	140.9 ± 22.1	< 0.001
Systems thinking *(7 items)*	22.6 ± 3.5	21.9 ± 3.3	< 0.001
Political leadership *(8 items)*	23.2 ± 6.1	20.1 ± 5.0	< 0.001
Collaborative leadership: building and leading interdisciplinary teams *(5 items)*	14.1 ± 3.8	12.6 ± 3.6	< 0.001
Leadership and communication *(7 items)*	19.7 ± 5.0	17.5 ± 4.0	< 0.001
Leading change *(6 items)*	18.1 ± 2.8	16.8 ± 3.2	0.005
Emotional intelligence and leadership in team-based organizations *(6 items)*	18.7 ± 3.6	16.9 ± 3.5	< 0.001
Leadership, organizational learning and development *(7 items)*	19.2 ± 4.0	17.6 ± 3.4	< 0.001
Ethics and professionalism *(6 items)*	18.6 ± 2.9	17.6 ± 2.7	0.018

* *Mann-Whitney test*.

There was evidence of a bigger gap in the leadership competency level in 2014 (mean value: 10.9; 95%CI = 8.0–13.9) compared with the year 2018 (mean: 2.9, 95%CI = 0.2–5.7) [Table 3]. The gap was the biggest for the “emotional intelligence and leadership in team-based organizations” domain (mean values: 0.2 in the year 2014 vs. −1.4 in the year 2018), followed by the “leadership and communication” subscale (2.7 vs. only 1.3, respectively) and next the “leadership, organizational learning, and development” domain (2.3 vs. 1.2, respectively).

**Table 3:** Gap in leadership competencies (difference in the summary scores between the *required* and the *current* level of competencies) among female health professionals in Albania (2014 vs. 2018).

**Domain (subscale)**	Mean values (95% CIs)*		*P*-value^*^
	**2014 sample (*N* = 105)**	**2018 sample (*N* = 121)**	
Gap in Leadership Competencies^*^			
Overall scale *(52 items)*	10.9 (8.0 to 13.9)	2.9 (0.2 to 5.7)	< 0.001
Systems thinking *(7 items)*	1.4 (1.0 to 1.8)	0.5 (0.1 to 0.9)	0.001
Political leadership *(8 items)*	1.2 (0.7 to 1.6)	0.8 (0.3 to 1.2)	0.221
Collaborative leadership: building and leading interdisciplinary teams *(5 items)*	1.9 (1.4 to 2.5)	1.2 (0.7 to 1.7)	0.070
Leadership and communication *(7 items)*	2.7 (2.1 to 3.4)	1.3 (0.7 to 1.9)	0.001
Leading change *(6 items)*	0.4 (−0.1 to 0.8)	−0.7 (−1.1 to −0.3)	< 0.001
Emotional intelligence and leadership in team–based organizations *(6 items)*	0.2 (−0.5 to 0.8)	−1.4 (−2.0 to −0.8)	0.001
Leadership, organizational learning and development *(7 items)*	2.3 (1.8–2.7)	1.2 (0.7–1.6)	0.001
Ethics and professionalism *(6 items)*	0.8 (0.5–1.2)	0.1 (−0.2 to 0.4)	0.002

* *Mean values, their respective 95% confidence intervals (95%CIs) and p-values from the General Linear Model*.

In crude (unadjusted) logistic regression models (Table 4), significant positive correlates of the gap in leadership competencies among Albanian female health professionals included age, work experience, employment in urban areas, middle, and especially high managerial job position/level and the first survey round (conducted in 2014). In multivariable-adjusted logistic regression models, there was evidence of a positive association between the gap in leadership competency level and: workplace in urban areas (OR = 3.2, 95%CI = 1.6–6.6); work experience (OR_[for 1 year increment]_ = 1.1, 95%CI = 1.0–1.2); first round of the survey conducted in 2014 (OR = 2.1, 95%CI = 1.0–4.3); and a high managerial job position/level (OR = 3.8, 95%CI = 1.6–9.3). On the other hand, upon simultaneous adjustment for all covariates, there was evidence of an inverse relationship with the age of female health professionals (OR_[for 1 year increment]_ = 0.9, 95%CI = 0.8–1.0) (Table 4).

**Table 4:** Correlates of the gap in leadership competencies among female health professionals in Albania.

**Variable**	**Crude (unadjusted) models**		**Multivariable-adjusted models^†^**	
	OR (95%CI)^*^	P^*^	OR (95%CI)^*^	P^*^
**Age *(1 year)***	1.03 (1.00–1.06)	0.038	0.92 (0.85–0.99)	0.032
**Place of Work:**				
Rural areas	1.00 (reference)	< 0.001	1.00 (reference)	0.001
Urban areas	3.41 (1.75–6.62)		3.22 (1.57–6.60)	
**Main Degree:**				
Health sciences	1.00 (reference)	0.789	1.00 (reference)	0.883
Other	0.91 (0.46–1.80)		0.94 (0.41–2.14)	
**Leadership Training:**				
No	1.00 (reference)	0.914	1.00 (reference)	0.251
Yes	1.03 (0.61–1.75)		1.50 (0.75–3.00)	
**Work experience *(1 year)***	1.05 (1.02–1.08)	0.001	1.12 (1.04–1.20)	0.004
**Job position:**		**0.017(2)**^‡^		**0.013(2)**^‡^
Low managerial level	1.00 (reference)	−	1.00 (reference)	−
Middle managerial level	1.68 (0.87–3.26)	0.125	1.71 (0.83–3.50)	0.143
High managerial level	3.08 (1.42–6.69)	0.004	3.80 (1.56–9.26)	0.003
**Year:**				
2018	1.00 (reference)	0.003	1.00 (reference)	0.052
2014	2.29 (1.33–3.95)		2.07 (0.99–4.33)	

* *Odds ratios (OR: competency gap vs. no competency gap), 95% confidence intervals (95%CIs) and p-values from the binary logistic regression*.

† *Adjusted simultaneously for all the variables presented in the Table*.

‡ *Overall p-value and degrees of freedom (in parentheses)*.

## Discussion

Main findings of this study conducted in a rapidly evolving society with tremendous public health challenges include a significant decrease in the leadership competency gap of female health professionals during the past 4 years (i.e., between the first survey round conducted in 2014 and the second round conducted in 2018). Furthermore, irrespective of the survey round/wave, independent positive correlates of the gap in leadership competency levels among female health professionals included their work experience, workplace in urban areas and, particularly, a high managerial job position.

In both survey rounds, there was evidence of a higher self-perceived level of the required leadership competencies than the current (actual) level of leadership competencies among female health professionals. Yet, the overall gap was considerably higher in the first round compared with the second round of the survey. Furthermore, in the first round of the study, all the eight subscale (domain) scores were significantly higher for the required competency level compared with the actual/current competency level, a finding which was not evident in the second round, where there was noted a negative score in the competency gap for the “leading change” and especially the “emotional intelligence and leadership in team-based organizations” subscales, indicating a “surplus” in the level of competencies for these two domains in the current job position of female health professionals in the year 2018.

Interestingly, regardless of the survey round, there was evidence of a positive relationship between the leadership competency gap and years of work experience of the Albanian female public health professionals. This may be explained by the fact that more experienced women are promoted to higher positions/levels which nevertheless require additional leadership skills and competencies. On the other hand, upon adjustment for work experience, there was evidence of an inverse association with age of participants indicating that older women perceived a lower gap in the leadership competencies compared with their younger counterparts, a finding which is seemingly intuitive. In addition, regardless of the job position/level, female professionals working in urban areas reported a significantly higher gap in leadership competencies, a finding which is expected given the higher pressure and demands for high-quality services in urban areas, especially in big cities. Surprisingly though, there was no significant association with previous leadership training courses, which points to the need for well-designed and carefully tailored training programs in the near future.

Czabanowska et al. have convincingly argued that a starting point in this process is to identify the competency capacities of future leaders in relation to population health and well-being and apply the study results to inform education, training, and culture change throughout the workforce (13). From this perspective and based on this valuable guiding principle, we considered that the description of the competencies supports the curriculum design and it can be used as a self-assessment instrument for students and public health professionals at all levels, helping, and supporting them to reflect and identify gaps in their knowledge, skills, abilities, and competencies (5, 12). It has been already pointed out that teaching of leadership is still uncommon in public health training programmes in most countries worldwide, particularly in those countries undergoing intensive public health reforms (16). Therefore, there is clearly an urgent need for a considerable investment in leadership training for public health professionals (17).

Regarding gender differences in general, the United Nations (UN) have included gender equality and women empowerment as Goal No. 5 in its list of Sustainable Development Goals (SGDs) for the 2030 Agenda (18). In turn, the European Union (EU) Progress Report of 2012 (19) has revealed several existing barriers regarding women leadership including different work conditions, unequal employment opportunities, lack of adequate networking, and unequal access to opportunities in general. While there is no reliable recent information about Albania, the evidence from other former communist countries indicates that women's participation in decision-making is very low, as illustrated in a fairly recent report from Ukraine (20). Furthermore, a pretty recent article indicates women in most countries as remarkably underrepresented in top leading positions an issue which, according to the authors of the report, may constitute a critical limitation toward organizational, societal and cultural progress regarding inclusion and balanced decision-making (21).

The current analysis may have several limitations including the study design, sampling strategy, and the information obtained. Regarding the possibility of selection bias, in both rounds of the study, a nationwide representative sample of public health professionals was included, which is reassuring. The response rate was very high in the first round of the survey conducted in 2014 (88%), but a bit lower in the second wave conducted in 2018 (81%), which may raise some concerns. As for the instruments of data collection, we employed an internationally standardized instrument (5), which had been previously validated in Albania (11). Also, in both rounds of the survey, the instrument used for the measurement of leadership competencies indicated very good internal consistency for both the required (desirable) and the actual/current leadership competency levels. On the face of it, there is no reason to assume differential reporting in the actual and/or required levels of leadership competencies among female professionals involved in the two rounds of the survey (2014 vs. 2018). Yet, the possibility of information bias cannot be completely excluded in such types of surveys. More importantly, findings pertinent to cross-sectional studies are not assumed to be causal and, therefore, should be carefully interpreted.

In conclusion, regardless of potential limitations, this analysis provides interesting and important evidence about the trends and selected correlates of the leadership competency level of female health professionals in Albania. It should be noted that the curriculum of undergraduate and/or postgraduate public health programs, as well as continuous professional education programs does not adequately foster leadership skills and competencies for future public health professionals and health workers in Albania (9, 12). Hence, findings from the current analysis should support policymakers and decision-makers in Albania for adapting, regulating and adjusting the public health curricula in accordance with the identified gaps and needs for further training, especially in the area of public health leadership. At a broader level, findings of this analysis should be confirmed and expanded in bigger representative samples of health professionals and public health specialists in different countries of the European region, with the ultimate goal of reinforcing the leadership skills and competencies among public health professionals at all levels of practice.

## Ethics Statement

This study was carried out in accordance with the recommendations of the University of Medicine, Tirana. The protocol was approved by the Department of Public Health, Faculty of Medicine, University of Medicine, Tirana, Albania.

## Author Contributions

KC, KaC, and GB contributed to the study conceptualization and design, analysis and interpretation of the data, and writing of the article. ER and HM commented comprehensively on the manuscript. All authors have read and approved the submitted manuscript.

### Conflict of Interest Statement

The authors declare that the research was conducted in the absence of any commercial or financial relationships that could be construed as a potential conflict of interest.
